# Federated Collaborative Learning with Sparse Gradients for Heterogeneous Data on Resource-Constrained Devices

**DOI:** 10.3390/e26121099

**Published:** 2024-12-16

**Authors:** Mengmeng Li, Xin He, Jinhua Chen

**Affiliations:** 1 College of Computer and Information Engineering, Henan University, Kaifeng 475001, China; limengmeng@henu.edu.cn; 2 Henan International Joint Laboratory of Intelligent Network Theory and Key Technology, Henan University, Kaifeng 475001, China; 3 College of Software, Henan University, Kaifeng 475001, China

**Keywords:** federated split learning, resource-constrained devices, heterogeneous data, sparse gradient, adaptive weight

## Abstract

Federated learning enables devices to train models collaboratively while protecting data privacy. However, the computing power, memory, and communication capabilities of IoT devices are limited, making it difficult to train large-scale models on these devices. To train large models on resource-constrained devices, federated split learning allows for parallel training of multiple devices by dividing the model into different devices. However, under this framework, the client is heavily dependent on the server’s computing resources, and a large number of model parameters must be transmitted during communication, which leads to low training efficiency. In addition, due to the heterogeneous distribution among clients, it is difficult for the trained global model to apply to all clients. To address these challenges, this paper designs a sparse gradient collaborative federated learning model for heterogeneous data on resource-constrained devices. First, the sparse gradient strategy is designed by introducing the position Mask to reduce the traffic. To minimize accuracy loss, the dequantization strategy is applied to restore the original dense gradient tensor. Second, the influence of each client on the global model is measured by Euclidean distance, and based on this, the aggregation weight is assigned to each client, and an adaptive weight strategy is developed. Finally, the sparse gradient quantization method is combined with an adaptive weighting strategy, and a collaborative federated learning algorithm is designed for heterogeneous data distribution. Extensive experiments demonstrate that the proposed algorithm achieves high classification efficiency, effectively addressing the challenges posed by data heterogeneity.

## 1. Introduction

In the industrial sector, IoT devices continuously collect production data and upload it to the cloud for model training. However, storing data in the cloud incurs significant transmission overhead and raises concerns about potential data privacy violations [1]. Federated learning (FL) has emerged as a promising approach in distributed machine learning, offering strong privacy protection while enabling collaborative learning [2]. In FL, the server sends the global model to participating devices, which train the model locally with their data. These IoT devices subsequently upload their updated model parameters to the server, where they are aggregated to refine the global model. This process is repeated iteratively until the global model reaches convergence [3]. Currently, many experts have conducted substantial research in this area. To improve the convergence speed of the model, Zhong et al. [4] designed an adaptive graph imputation generator to explore the potential links between subgraphs and combined the multi-function evaluator with the negative sampling mechanism to explore the global information flow to construct a multi-edge collaborative SpreadFGL framework. In addition, to improve the utilization efficiency of resources, Chen et al. [5] used proximal items to improve classical deep reinforcement learning and avoided the problem of action dispersion by reducing the variance of action–value estimation by decreasing the frequency of network updates, to solve the problem that the existing resource allocation cannot adapt to the personalized dynamic environment. Therefore, they designed a computational offloading and resource allocation method based on personalized deep reinforcement learning to achieve a higher success rate of task execution in different scenarios. To ensure the robustness of the collaborative system, Chen et al. [6] considered the differences between multi-dimensional user characteristics, activities, and memory to achieve more accurate content recommendations, which is by considering the inefficient collaboration between multi-edge nodes and the risk of being attacked in the collaborative caching mechanism. To mitigate the issue of posterior collapse, they proposed the Discrete-Categorical Variational Auto-Encoder and designed a robust federated deep learning framework. These methods enhance the cooperation efficiency of edge devices from different perspectives. Additionally, it is important to note that in distributed training frameworks, the server typically deploys small-scale model structures on clients, which can lead to some prediction errors [7]. To enhance performance in distributed applications, increasing the complexity and parameters of deep learning models is often a practical solution [8]. However, IoT devices are often constrained by limited memory, storage, and computing resources, hindering their ability to train large-scale models locally [9,10]. Moreover, transmitting large-scale model parameters between clients and servers can introduce substantial communication delays [11].

To train high-performance models on resource-constrained devices, federated split learning (FSL) [10] splits model training between clients and servers. FSL divides the model into client and server components by cutting layers, shifting part of the training to the resource-rich server [11]. This approach reduces the client’s computational load, enabling the deployment of large models on lightweight devices and improving training efficiency [12]. For instance, in the SplitFed framework [13], clients perform forward propagation on their models using local data and upload the labeled intermediate output to the server. The server then performs forward and backward propagation on its model and sends the gradients back to the client, which completes backpropagation and sends its part of the model to the server for aggregation [10]. However, the client and server need to communicate with each other when the model is updated, resulting in high network overhead. He et al. [14] introduced a training strategy based on local loss, replacing the traditional global loss function to train the model, so that the client model can be updated without accepting backpropagation from the server model. While this approach reduces network overhead, it heavily depends on the server’s computing resources. Nguyen et al. [11] divided the large-scale deep learning model into a set of several small sub-models, trained them in parallel on multiple devices, and designed a framework that allowed multiple client clusters to cooperate. This allowed clients within the same cluster to learn from each other, improving the overall model performance. However, during the training process, all devices in the cluster interact with a large number of model parameters, resulting in high communication costs. The direct way to reduce communication costs is by compressing the data exchanged within the FL framework through compression processing. The sparse gradient strategy reduces the amount of data transmitted during each communication without significantly reducing the accuracy [15,16]. Thus, sparse gradients can improve training efficiency and resource utilization on edge devices. For example, Thonglek et al. [15] calculated the absolute difference between the parameters of the local model before and after training, determined the upper quantile of the exchange of updated parameters between the client and the server, and reduced the communication cost. Sun et al. [16] introduced the Top-k sparsification method into the secure aggregation protocol, which reduced the privacy risk and the communication overhead. Therefore, inspired by the above literature, this paper proposes a sparse gradient strategy to reduce communication data and introduces dequantization to minimize the loss of model accuracy.

Although FSL demonstrates commendable performance under resource constraints, it faces significant challenges due to data heterogeneity [17]. In real-world scenarios, the diverse data sources from IoT devices often result in substantial distributional discrepancies among clients, leading to significant heterogeneity in the datasets [18,19]. This heterogeneity can cause gradient divergence, which adversely affects the performance of the aggregated global model [8]. To reduce the impact of non-independent and identically distributed (non-IID) data on model performance, many existing solutions, such as local normalization, are applied to data-sensitive layers [20]. However, this approach, while effective in reducing data discrepancies, hinders knowledge sharing between clients, which limits the overall model’s performance and collaborative potential. To improve collaboration among clients while addressing data heterogeneity, the similarity evaluation mechanism is introduced to encourage clients with similar data distributions to train the model collaboratively [21,22]. Although this approach enables personalized models for clients, it may increase the imbalance between clients and overlook the influence of client models on the global model. To tackle this issue, this paper measures the difference between the local model and the global model using Euclidean distance and applies the adaptive aggregation weight to optimize the overall performance and efficiency of the federated learning system. This paper aims to reduce communication overhead, address data heterogeneity, and improve the training efficiency of resource-constrained devices. To achieve this, we propose a novel federated collaborative framework that combines sparse gradient strategies with adaptive aggregation weight. This combination improves the global model’s accuracy and training efficiency without compromising individual clients’ specific performance. First, the sparse gradient strategy is introduced to reduce the communication required for each update between clients. Second, the adaptive aggregation weight strategy is designed to mitigate the impact of data heterogeneity on model performance. Finally, these strategies are integrated into the federated collaboration framework to ensure the robustness of the training process.

The main contributions of this paper are as follows:

(1) To address the issue of high communication costs in existing federated collaboration frameworks, the sparse gradient strategy based on position Mask is designed to reduce data transmission. At the same time, gradient dequantization is introduced to restore the original dense gradient tensor, minimizing the negative impact on model performance while maintaining communication efficiency.

(2) To deal with the challenge of data heterogeneity between clients, an adaptive aggregation weight strategy based on the Euclidean distance is proposed. According to the difference between the local model of the client and the global model, the weight of the client is dynamically adjusted, which reduces the impact of data heterogeneity and enhances the collaboration between the clients.

(3) To enable resource-constrained devices to efficiently participate in distributed training, the new federated collaboration framework based on sparse gradient is designed. This framework not only reduces communication overhead but also improves the efficiency of collaborative training tasks for resource-constrained devices.

The remainder of our work can be arranged as follows: Section 2 describes the related work. Section 3 describes the traditional FL framework, federated divide, and collaborative framework. Section 4 introduces the sparse gradient quantization strategy and the adaptive weighting strategy and presents the novel federated collaborative framework. Section 5 presents the experimental results. Finally, Section 6 concludes the article with a summary.

## 2. Related Work

### 2.1. Federated Split Learning

To analyze the massive data in the production process, complex models are trained to reduce production risks [23]. However, many mobile devices are limited by resources and cannot handle local training tasks, resulting in model training failures. To address this, federated split learning (FSL) migrates part of the trained models from the device side to the server side, enabling the application of deploying large-scale models on lightweight devices [12]. Thapa et al. [13] introduced the SplitFed framework, where both client and server independently update their respective models, and the server aggregates these updates to create a global model. Tian et al. [24] deployed the computationally intensive Transformer layer on the server and proposed a novel framework for pre-training language models while training the embedding and header layers on the client side, enabling parallel task training across multiple clients. Kortoçi et al. [25] extended the FSL framework to a distributed collaborative learning framework that generates adversarial networks, which is by placing the generator model on the server and training a discriminator network with client data. Despite these advancements, each local update in these methods requires communication between client and server, which leads to high network overhead. To address this, Luo et al. [26] designed a novel FSL framework utilizing mutual knowledge distillation, which supports personalized local models and offers cost-effective solutions for model training and device selection. He et al. [14] proposed a training strategy based on local loss functions, allowing client models to update independently without server-side backpropagation, thereby reducing communication costs. Although these methods decrease network overhead, they still rely heavily on server computing resources. Nguyen et al. [11] divided a large model into multiple smaller sub-models, enabling parallel training across devices and a collaboration framework in which multiple client clusters share model improvements, enhancing overall model performance. However, all devices in the cluster need to interact with model parameters during training, which extends training time. Qin et al. [23] employed split learning to transfer the training workloads from devices to the cloud, leveraging the cloud’s superior computing power and compressing data to reduce both computational and communication costs. To reduce the transmission traffic, this paper designs an efficient federated collaborative learning framework.

### 2.2. Heterogeneous Federated Learning

FL faces a serious class imbalance between client data, which may lead to a large gap between local and global models, resulting in a poorly performing global model. Therefore, designing an efficient collaboration strategy to handle heterogeneous data remains a challenging task. To address these challenges, Wang et al. [20] constructed the FedNova to eliminate the target inconsistency, which normalized the local model parameters in each iteration and averaged them on the server. This approach enables clients to perform varying numbers of local updates. Huang et al. [21] calculate the personality weight between clients by adopting the attention message-passing mechanism, which encourages collaboration among clients with similar data characteristics. Liu et al. [22] leveraged neural network sparsity to generate data representations and used Euclidean distance to measure data distribution similarity, thereby achieving more personalized results. While these approaches enhance personalized model performance by fostering collaboration among clients with similar data distributions, they can inadvertently intensify existing data imbalances [27,28]. Wu et al. [29] designed a FedAdp framework, which measured the contribution of participating devices according to the gradient information from local clients and then adaptively assigned different weights during communication rounds to optimize global model aggregation. Jiang et al. [30] employed an attention mechanism to capture correlations among local features, generating aggregation weights based on model differences. They further designed a personalized federated learning algorithm using a multi-head attention mechanism to enhance personalized learning. FSL also remains challenged by data heterogeneity [17], inspired by the above results, an adaptive weight aggregation strategy is designed for heterogeneous data between clients.

## 3. Preliminaries

### 3.1. Traditional FL Framework

Each client in FL uses the local dataset for local training, aggregating the model parameters on the server. We consider *K* clients participating in the training, indexed by *K* = {1, 2, ⋯, *K*}. Each client *k* updates its local model weight *w_k_* by using the local data *D_k_*, aiming to enhance the performance of the global model. Thus, the objective of FL can be formulated as follows [21]:

(1) $$\underset{w}{\min}l(f(w;x),y)=\sum_{k=1}^{K}\frac{\left|D_{k}\right|}{\left|D\right|}l(f(w_{k};x),y),$$

where l(·) is the loss function, *w* denotes the global model weight parameter, *w_k_* denotes the model weight parameter of client *k*, and *f*(·) represents the model architecture. The dataset *D_k_* = (*x_k_*, *y_k_*) represents the client’s local data, where *x* is the input data, and *y* is the corresponding label.

After local training, each client sends its finally updated local model *w_k_* to the server. The server then updates the global model parameters by performing a weighted average aggregation [31], as follows:

(2) $$W^{t+1}=\frac{1}{\left|D\right|}\sum_{k=1}^{K}|D_{k}|w_{k}.$$

When the next communication round begins, the server transmits the updated global model to all clients. This process continues until either the preset number of communication rounds *T* is reached or the model performance meets the requirements.

### 3.2. Federated Divide and Collaborative Framework

The traditional FL framework typically assumes that all participating devices can independently train the whole ML model. However, due to the resource limitations on lightweight edge devices, such as computing power, communication, and memory resources of clients, it is often unable to undertake the task of training large-scale network models. Therefore, a novel federated divide and collaborative framework has been proposed to support the training of complex convolutional neural networks on resource-constrained devices, as discussed in reference [11].

Before the training begins, a subset of *S* clients {*c*_1_, *c*_2_, …, *c_S_*} is randomly selected from *K* devices to form cluster *C*. The original model *W* is then divided into *S* sub-model sets *W_en_* = *E*{*W*_1_, *W*_2_, …, *W_S_*} according to the network parameters in each cluster *C*∈ℂ. Next, each sub-model *W_i_* is split using the cutting layer: the lower sub-model, which extracts abstract representation of the input data, and the upper sub-model, which is responsible for prediction. The server then sends each part of the global model to the participating clients, enabling devices within each cluster to update the ensemble model in parallel using only their local data.

During the training process, the first client in each cluster *C*∈ℂ is designated as the main client, while the others act as proxy clients. The server sends $W_{en}^{m}=E\{W_{1}^{m},W_{2}^{m},\ldots ,W_{s}^{m}\}$ to the main client in each cluster and distributes $W_{i}^{p}$ (*i* = 1, …, *S*) to each *i*-th client. The main client’s sample data are augmented to produce *S* versions, which are then passed in $\left\{W_{1}^{m},W_{2}^{m},\ldots ,W_{s}^{m}\right\}$ for forward propagation, resulting in *S* activated $a(W_{i}^{m})$. Then, the main client sends the activation $a(W_{i}^{m})$ of *S* cutting layers to *S* − 1 proxy clients, while retaining one abstract representation. Each client *c_i_*∈{*c*_1_, *c*_2_, …, *c*_S_} uses the activation data $a(W_{i}^{m})$ to perform forward propagation on its corresponding upper sub-model and then sends its prediction result *p_i_* to the server. The server then collects the predictions {*p*_1_, *p*_2_, …, *p*_s_} from all devices, designs regularization terms by using Jensen–Shannon divergence, and designs loss functions by combining cross-entropy classification, facilitating mutual learning among devices. The loss calculated by the server is sent to each client, where it is backpropagated to each corresponding sub-model’s cutting layer. The gradient at the cutting layer are sent to the main client, which updates after completing backpropagation. Finally, the main client transfers the update to the next designated main client, and this process continues until every client in the cluster has completed training as the main client.

After training, the last main client *c*_S_ in each cluster *C* sends the updated ensemble model $W_{C}^{m}$ of the global lower sub-model $W_{en}^{m}$ to the server, while each device *c_i_* sends its updated upper sub-model $W_{i}^{p}$ to the server, forming the upper ensemble model $W_{C}^{p}=E\{W_{1}^{p},W_{2}^{p},\ldots ,W_{s}^{p}\}$. The server then merges $W_{C}^{m}$ and $W_{C}^{p}$ to create an ensemble model *W_C_* for each cluster. Finally, the server aggregates the ensemble models from all clusters to obtain a new global ensemble model.

## 4. Efficient Federated Collaborative Learning Methodology

**Figure 1:**
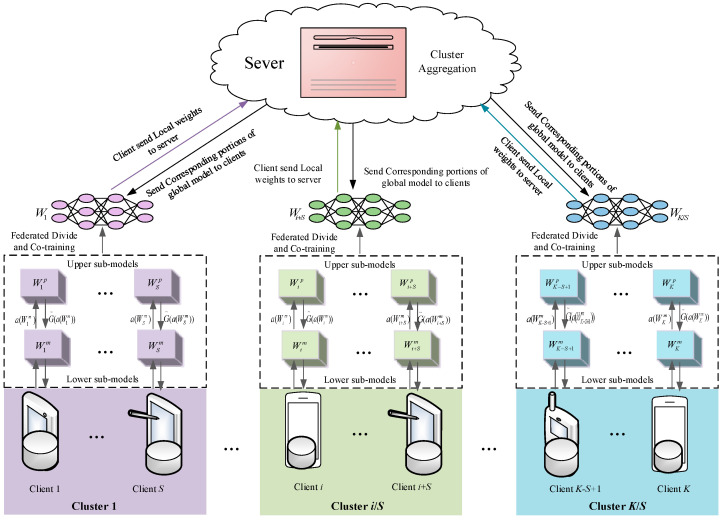
Overall framework of our designed FedCS model.

To address the problem of heterogeneous data in resource-constrained clients, this paper provides a federated collaborative learning with sparse gradients framework (FedCS), as shown in Figure 1.

The main steps are as follows: Firstly, in each cluster, the model parameters are divided into *S* sub-model sets, and the sub-models are divided into lower and upper sub-model by using the cutting layer. The server distributes the lower sub-model parameters to the main client and the upper sub-model parameters to all clients. Secondly, the main client uses its local data to perform forward propagation on the lower sub-model up to the cutting layer and sends the activation values of this layer to each client. Each client then propagates the activation values through its upper sub-model. Then, each client sends its predicted values back to the server, which calculates the loss function and returns the gradients to each client. Each client performs backpropagation on its network, and after sparse processing, sends the sparse gradient to the main client. The main client receives the sparse gradients and uses the gradient dequantization strategy to recover the original gradient. Then, it performs backpropagation on the upper sub-model and sends it to the next main client. After all devices in the cluster have been trained as the main client, the network models of all devices are sent to the server. Then, the server integrates the upper sub-model using the adaptive weighting strategy, and combines them with the lower sub-model to form the model for the cluster. Finally, the server performs weighted averaging across clusters to generate an updated global model. This training process continues until the specified stopping criteria are met.

### 4.1. Sparse Gradient Quantization Strategy

Within the framework of federated collaborative learning, gradient or model parameters must frequently be synchronized between each client (i.e., proxy client) and the main client. Given the large size of gradients or parameters in large network models, training such models on resource-constrained clients can lead to significant communication and computational costs. To address this, the sparse gradients are employed to retain only the most important gradient elements, thereby reducing both computation and communication overhead [32]. While the sparse gradients help alleviate these costs, it is essential to carefully balance them to minimize overhead without compromising model accuracy. To further mitigate accuracy loss, the gradient dequantization is used to save storage space and computing resources while preserving model performance as much as possible. By integrating sparse gradients with gradient dequantization, an efficient communication strategy for large-scale distributed training is developed, optimizing both performance and resource usage.

In each communication round, the server transmits part of the global ensemble model to the respective devices in each cluster. The main client uses data augmentation to obtain *S*-enhanced versions of the sample, and pass them to $\left\{W_{1}^{m},W_{2}^{m},\ldots ,W_{s}^{m}\right\}$. And it obtains *S*-cutting layer activations $a(W_{i}^{m})$ and sends the activations $a(W_{i}^{m})$ to *S* − 1 proxy clients for forward propagation. Following the approach of reference [11], the server collects the predictions {*p*_1_, *p*_2_, …, *p*_s_} of all devices, and uses Jensen–Shannon divergence to design regularization terms, combining it with cross-entropy classification to design loss functions, which is calculated as follows:

(3) $$L_{\cot}\left(p_{1},p_{2},\ldots ,p_{S}\right)=H\left(\frac{1}{S}\sum_{k=1}^{S}p_{k}\right)-\frac{1}{S}\sum_{k=1}^{S}H\left(p_{k}\right),$$

(4) $$L^{\left(C\right)}(W_{en})=\sum_{i=1}^{S}L_{ce}\left(p_{i},y_{1}\right)+\lambda_{\cot}L_{\cot}\left(p_{1},p_{2},\ldots ,p_{S}\right),$$

where *p_i_* (*i* = 1, …, *S*) represents the $W_{i}^{p}$ output vector of client *c_i_*, $L_{\cot}$ is the cooperative training loss, *λ_cot_* is the weight factor, and $L_{ce}$ is the cross entropy classification loss. The combined loss $L^{\left(C\right)}(W_{en})$ is then sent to all devices, where each devices performs backpropagation on its upper sub-model.

When backpropagating to the cutting layer, the high-dimensional gradients need to be transmitted to the main client, a process that requires significant time costs. Given the gradient tensor *G* = (*g*_1_, *g*_2_, …, *g_n_*) of the model, the sparse gradient can be expressed as follows:

(5) $$\tilde{G_{i}}=\left\{\begin{array}{l} g_{i}\;\mathrm{if}\;|g_{i}|\geq \tau \\ 0\;\mathrm{if}\;|g_{i}|<\;\tau \end{array}\right.,$$

where *g_i_* represents the *i*-th element in the gradient vector, and τ represents the sparse threshold. To record which gradient values are retained or zeroed, a Mask needs to be generated, which is defined as follows:

(6) $${\tilde{G}}_{sparse}=\tilde{G_{i}}\times Mask,$$

where *Mask* is a Boolean mask that filters out partial gradients greater than the sparse threshold. According to Equation (6), only those gradient elements whose absolute value is greater than or equal to τ are retained, and the rest are set to zero. The main client receives the sparse gradient from the cutting layer, and at the same time, it needs to dequantize the sparse gradient and restore the original dense gradient tensor:

(7) $${\tilde{G}}_{Deq}={\tilde{G}}_{sparse}\times Mask,$$

where *Mask* ensures that only the sparse gradient terms are involved in the operation.

### 4.2. Adaptive Weighting Strategy

Due to the significant variation in data distribution across clients, simply applying a weighted average for aggregating client models on the server can overlook each client’s unique contributions, leading to the suboptimal performance of the global model. To address this issue, the server calculates the Euclidean distance between each client’s local model and the global model after training. This distance serves as an evaluation of each client’s performance in the classification task. Based on these distances, the server dynamically assigns aggregation weights to clients, thereby refining their influence on the global model update. This adaptive weighting approach improves the FL system’s overall performance and efficiency.

After each training round, the final main client *c*_S_ in each cluster *C* sends the updated ensemble version $W_{C}^{m}$ of the global sub-model $W_{en}^{m}$ to the server. Simultaneously, each device *c_i_* in the cluster sends its updated upper sub-model $W_{i}^{p}$ to the server, which then constructs the ensemble model $W_{C}^{p}=E\{W_{1}^{p},W_{2}^{p},\ldots ,W_{s}^{p}\}$. During the ensemble process, the server calculates the Euclidean distance between each client model and the global model, and the weights are allocated based on this distance:

(8) $$w_{i}=\frac{\sum_{k}{\left\|\theta_{g}^{\left(k\right)}-\theta_{i}^{\left(k\right)}\right\|}_{2}}{\sum_{i=1}^{n}\left(\sum_{k}{\left\|\theta_{g}^{\left(k\right)}-\theta_{i}^{\left(k\right)}\right\|}_{2}\right)},$$

where $\theta_{g}^{\left(k\right)}$ and $\theta_{i}^{\left(k\right)}$ represent the *k*-th parameters of the global model and the client model, respectively, and ${\left\|\cdot \right\|}_{2}$ represents the L2 norm. The aggregation of the proxy client model adopts a weighted average method, and the weights are determined by the previous adaptive weights:

(9) $$\theta_{g}=\frac{\sum_{i=1}^{n}w_{i}∙\theta_{i}^{\left(k\right)}}{\sum_{i=1}^{n}w_{i}},$$

where *w_i_* is the weight of client *i*, $\theta_{g}$ is the global model parameter, and $\theta_{i}^{\left(k\right)}$ is the *k*-th parameter of the *i*-th proxy client. The server merges $W_{C}^{m}$ and $W_{C}^{p}$ to create an ensemble model *W_C_* for each cluster. It then aggregates the ensemble models from all clusters to obtain a new global ensemble model.

(10) $$W_{en}=\frac{1}{N}\sum_{C\in \mathbb{C}}N^{\left(C\right)}W_{C},$$

where *N*^(*C*)^ is the cardinality of cluster *C*, representing the number of clients in the cluster.

### 4.3. FedCS Algorithm

In what follows, we design the novel federated collaborative framework for heterogeneous data on resource-constrained devices (FedCS), detailed in Algorithm 1. Before training begins, for each cluster *C*∈ℂ containing *S* devices, the original model *W* is divided into *S* sub-models according to the network parameters. Each sub-model *W_i_* is further divided into two parts at the cutting level. The server assigns these sub-models to the participating clients for training, with clients in each cluster updating their models in parallel using local data.

In the *r*-round training process, the first device in the cluster is treated as the main client. The main client applies data augmentation to its sample, passing it through the sub-model $W_{en}^{m}=E\{W_{1}^{m},W_{2}^{m},\ldots ,W_{s}^{m}\}$ to obtain the activation $a(W_{i}^{m})$ of *S* cutting layers. Next, the main client sends the activation $a(W_{i}^{m})$ to *S* − 1 proxy clients in the cluster. Each proxy client *c_i_*∈{*c*_1_, *c*_2_, …, *c*_S_} completes the forward propagation of the upper sub-model in parallel, thus obtaining the predicted value of the sample. The server then collects the predictions {*p*_1_, *p*_2_, …, *p*_s_} from all devices, calculates the losses using a designated loss function, and propagates gradients back to the cutting layer in each client’s sub-model. Each client applies the sparse gradient strategy according to the *Mask* and sends the sparse gradient to the main client. The main client then performs gradient dequantized, updates the sub-models $W_{en}^{m}=E\{W_{1}^{m},W_{2}^{m},\ldots ,W_{s}^{m}\}$ via backpropagation, and sends the updated $W_{en}^{m}$ to the next main client in sequence. This process continues until each device in the cluster has taken its turn as the main client.
**Algorithm 1:** FedCS**Input:** the set of all devices *S*, the batch size *B*, the number of global rounds *R*, the model of cluster *C*∈ℂ
*W_C_*, the number of data samples *N*(*C*), the cutting layer *L*, the learning rate *η*
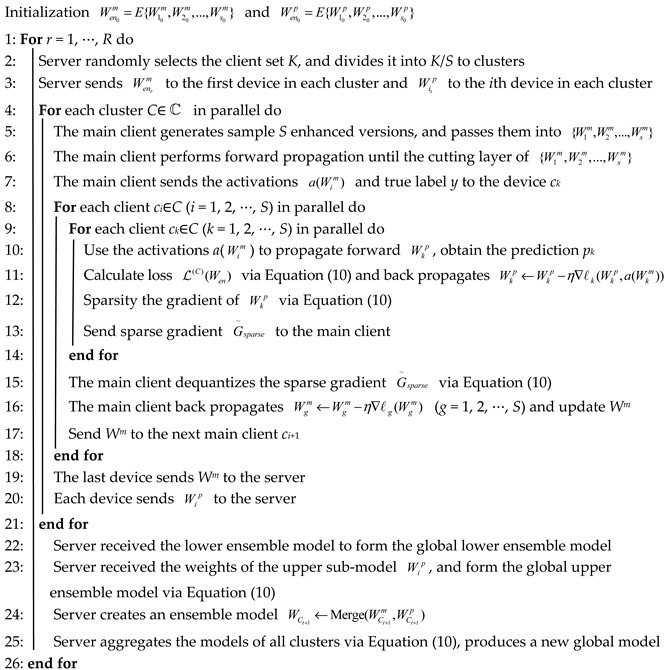


After the *r*-th training round, the main client sends the updated ensemble version $W_{C}^{m}$ of the global lower sub-model $W_{en}^{m}$ to the server. Each device *c_i_* sends its updated upper sub-model $W_{i}^{p}$ to the server, which then applies an adaptive weight strategy to obtain the upper ensemble model $W_{C}^{p}=E\{W_{1}^{p},W_{2}^{p},\ldots ,W_{s}^{p}\}$. The server merges $W_{C}^{m}$ and $W_{C}^{p}$ to create an ensemble model for each cluster, and aggregates the ensemble models from all clusters using a weighted average to generate a new global ensemble model.

## 5. Experiments

### 5.1. Experiment Preparation

**Figure 2:**
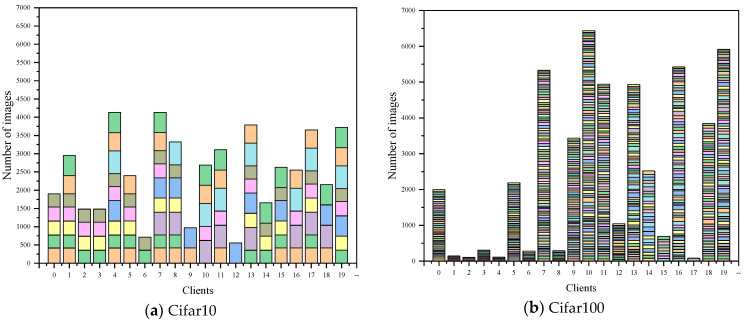
The QS distribution information of samples and labels for two datasets on 20 clients.

To evaluate the classification performance of the designed algorithm, we conducted a series of experiments on the Cifar10 [33] and Cifar100 [33] datasets. The Cifar10 dataset consists of 60,000 color images from 10 different classes, including 50,000 training images and 10,000 test images, with a resolution of 32 × 32 pixels. The Cifar100 image dataset collects 100 classes of images, each containing 500 training images and 100 test images, also at a resolution 32 × 32 pixels. Following the method described in [31], we randomly used 80% of the images for training and the remaining 20% for testing. To simulate heterogeneous data conditions on clients, we randomly assigned the samples and labels to multiple clients. Each client can hold varying quantities of classes and samples, known as quantity skew (QS), and the specific data distribution information is shown in Figure 2. In addition, to illustrate the performance of different sizes models on resource-constrained devices, we evaluated the WRN-16-8 [34], ResNet50 [35], and ResNet110 [35] models on the selected datasets. The ACC metric, as discussed in [11,31], was used to demonstrate and analyze the classification performance of all comparative methods, with the best values highlighted in bold.

To evaluate the performance of the proposed algorithm, FedCS is compared with FedAvg [31], FedProx [36], SplitFed [13], and FedDCT [11] under the same training strategy. To ensure fairness in the experiment, the same training methods and hyperparameter settings as [11,13,31,36] are adopted, and the initial parameters of the model are shown in Table 1. The weights are initialized using the Kaiming initialization technique [37], and the initial learning rate *η*_0_ = 0.1. The network is trained using the Nesterov-accelerated SGD optimizer to train the network with a momentum of 0.9. During the training phase, common data enhancement techniques are also used, including random cropping, random flipping, normalization, random erasure, mixing, and the RandAugment method [31,38]. All experimental configurations are running on Windows 10, with an Intel i5 CPU (3.20 GHz), manufactured by Intel Corporation, Santa Clara, CA, USA, 8.0 GB of RAM, and an NVIDIA GeForce GTX 3090 GPU, manufactured by NVIDIA Corporation, Santa Clara, CA, USA, 64 GB of RAM, and all codes are executed on PyTorch 3.9. 

### 5.2. Efficiency Evaluation

**Figure 3:**
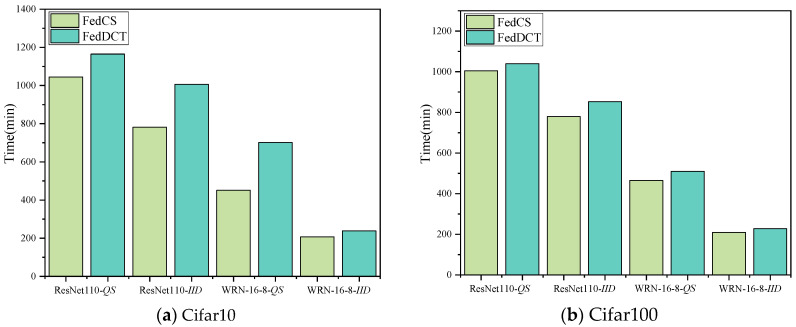
The runtime results of the two datasets under four experimental scenarios.

To evaluate the running efficiency of FedCS, we compare our method with state-of-the-art approaches on the Cifar10 and Cifar100 datasets. Additionally, to verify the effectiveness of FedCS under different environments, two types of data distributions are implemented on the clients: 1. quantity skew (QS), which simulates the imbalance in client data distribution, and 2. independent and identically distributed (IID) data. Both network models are deployed on 20 clients for evaluation. Two network models were employed to demonstrate performance under these conditions. Since the operation of FedAvg, FedProx, and SplitFed methods is highly dependent on the server, our method follows the same training approach as FedDCT, which does not rely on the server. Consequently, to present a fair evaluation of algorithmic efficiency, we focused on comparing FedCS and FedDCT. The experimental results are shown in Figure 3. As illustrated in Figure 3, for the two data distributions of Cifar10 and Cifar100 datasets, the running time of FedCS is shorter than FedDCT. This indicates that the running efficiency of FedCS surpasses that of FedDCT. In summary, by leveraging a sparse gradient strategy, FedCS significantly reduces data transmission during gradient updates in distributed training, thereby accelerating model parameter updates and shortening the overall training duration.

### 5.3. Ablation Experiment

**Figure 4:**
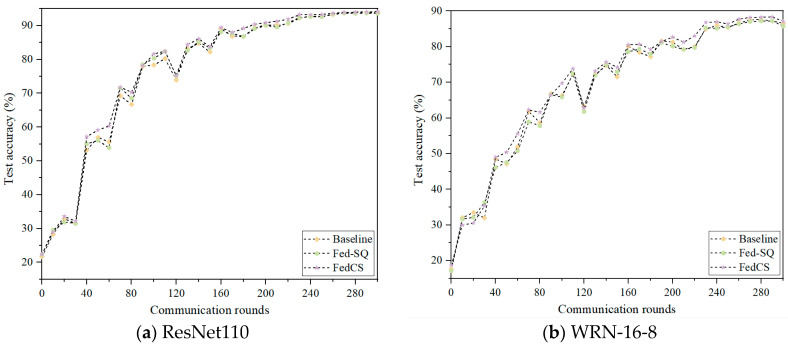
Classification performance of different components on 20 clients.

**Figure 5:**
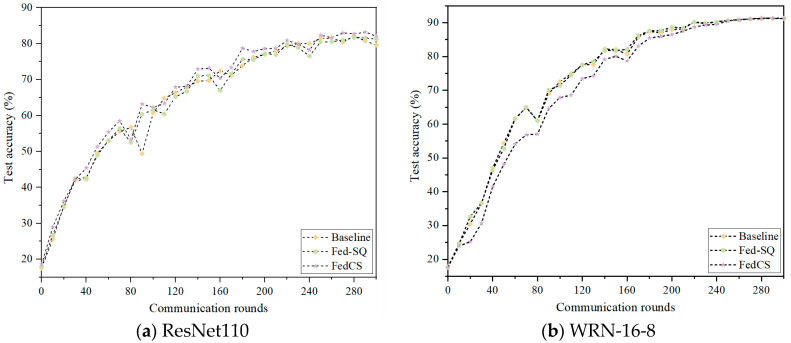
Classification performance of different components on 40 clients.

To validate the effectiveness of the strategy proposed in this paper, experiments are conducted on the Cifar10 dataset using 20 clients and 40 clients with QS distribution. The framework from reference [8] is employed as the baseline algorithm, with Sparse Gradient Quantization Strategy used as Fed-QS, compared against our algorithm FedCS. The results of these experiments using two network models are depicted in Figure 4 and Figure 5. In these Figures, the abscissa represents the number of rounds of communication, and the ordinate represents the accuracy of the rounds of communication. According to Figure 3, when applying both models to 20 clients, FedCS consistently achieved optimal performance. Additionally, Fed-QS using Sparse Gradient Quantization Strategy demonstrated relatively strong performance compared to the baseline algorithm. In each training round, although Fed-QS’s classification accuracy is not the highest, it remains close to the baseline algorithm. As shown in Figure 4, when applying the ResNet110 model on 40 clients, FedCS consistently achieves optimal performance. Fed-QS, using the Sparse Gradient Quantization Strategy, shows a classification performance comparable to the baseline algorithm. When applying the WRN-16-8 model on 40 clients, Fed-QS using Sparse Gradient Quantization Strategy also shows classification performance comparable to the baseline algorithm. However, FedCS shows slower convergence speed during training compared to 20 clients. This phenomenon is attributed to the evaluation of differences between client and server models during aggregation. Combining the efficiency evaluation, the experimental results show that the Sparse Gradient Quantization Strategy can reach the same level as the uncompressed model in terms of convergence speed and final accuracy.

### 5.4. Classification Results

**Figure 6:**
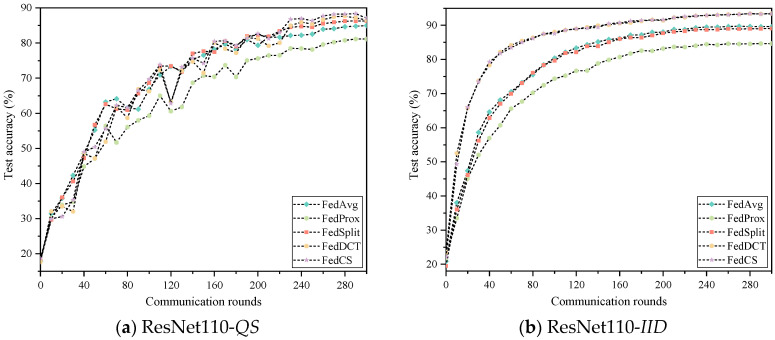

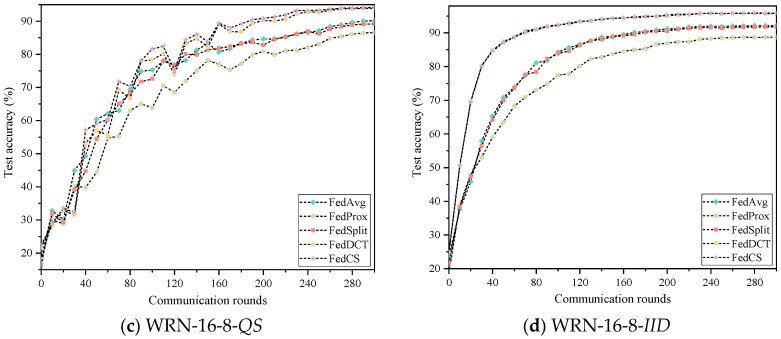
Experimental results of five algorithms on the Cifar10 dataset on 20 clients.

**Figure 7:**
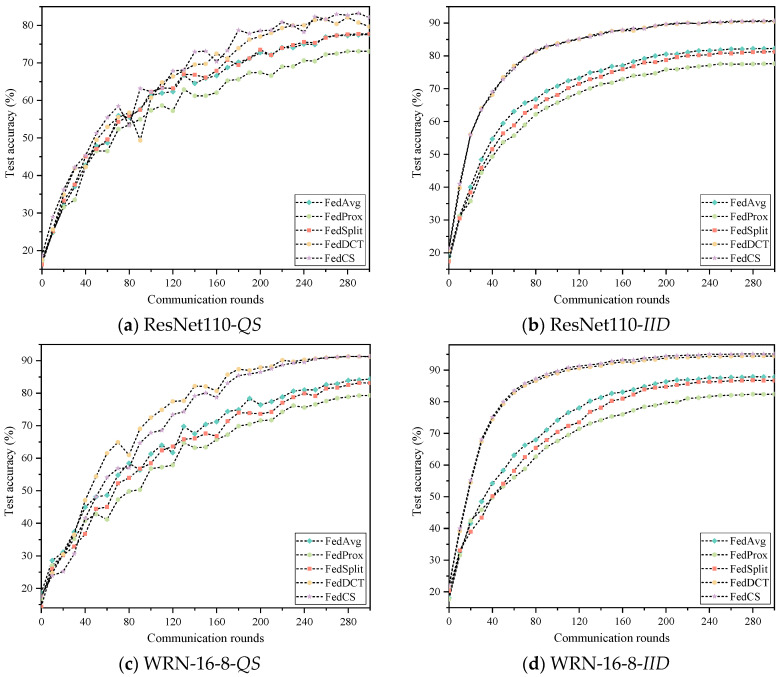
Experimental results of five algorithms on the Cifar10 dataset on 40 clients.

**Figure 8:**
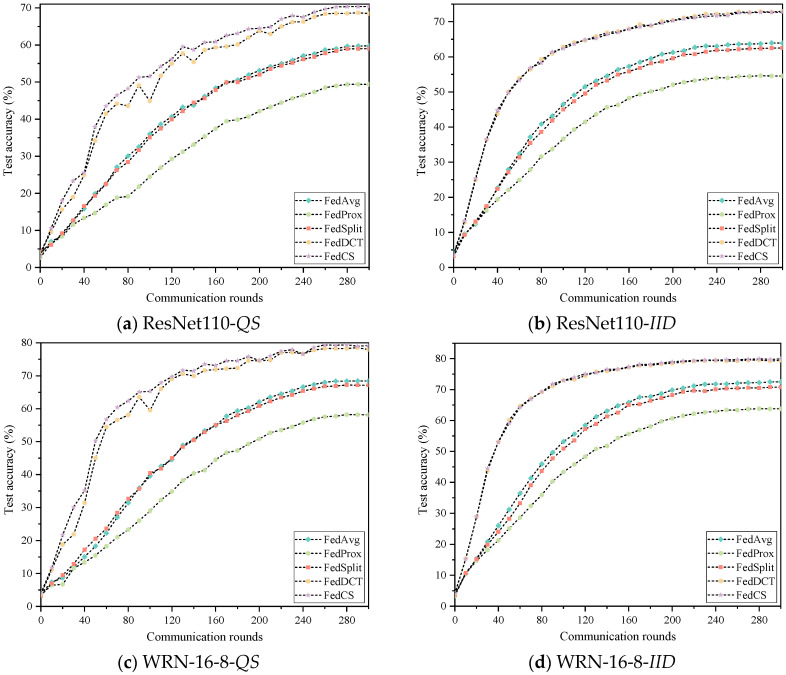
Experimental results of five algorithms on the Cifar100 dataset on 20 clients.

**Figure 9:**
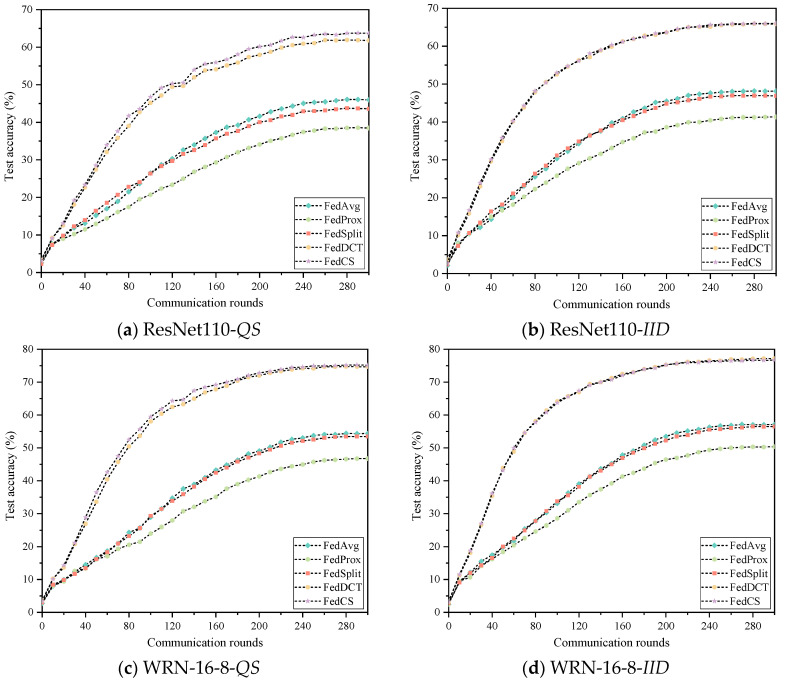
Experimental results of five algorithms on the Cifar100 dataset on 40 clients.

To evaluate the effectiveness of FedCS, we compared it with the latest state-of-the-art FL methods, including FedAvg [26], FedProx [18], SplitFed [10], and FedDCT [8], on the Cifar10 and Cifar100 datasets. Furthermore, to simulate distributed environments at different scales, we randomly assigned Cifar10 and Cifar100 datasets to either 20 or 40 clients, respectively, and employed two network models for demonstration. The experimental results for each scenario are presented in Table 2 and Table 3 and Figure 6, Figure 7, Figure 8 and Figure 9. In these Figures, the abscissa represents the number of rounds of communication, and the ordinate represents the accuracy of the rounds of communication. As seen in Table 2, FedCS achieves excellent performance in different experimental scenarios. To be specific, for the case with 20 clients, the ResNet110 model is first deployed on the clients. When dealing with the data distribution of *QS*, FedCS is 0.53–5.93 higher than other comparison algorithms. The FedCS is 0.05 lower than FedDCT but 3.79–7.2 higher than other algorithms when dealing with *IID* data distribution. Secondly, the WRN-16-8 model is deployed on the client. When dealing with the data distribution of *QS*, FedCS is 0.27–7.55 higher than other comparison algorithms. The FedCS is 0.05 lower than FedDCT, but 3.69–7.07 higher than other algorithms when dealing with *IID* data distribution. For the case with 40 clients, the ResNet110 model is first deployed on the client. The FedCS is 2.41–8.97 and 0.24–13.08 higher than other comparison algorithms when dealing with *QS* and *IID* data distribution. Secondly, when deploying WRN-16-8 model on the client, FedCS is 0.03–11.96 and 0.68–12.73 higher than other comparison algorithms when dealing with *QS* and *IID* data distribution. Additionally, the performance of the algorithm on 20 clients is better than that on 40 clients. Moreover, as shown in Figure 4 and Figure 5, FedCS surpasses the FedDCT algorithm in most cases, but it is always higher than the FedAvg, FedProx, and FedSplit algorithms. Above all, FedCS demonstrates strong results in dealing with *QS* data distribution, indicating its effectiveness in mitigating the issue of heterogeneous data.

From Table 3, we observe that FedCS can achieve excellent performance across various experimental scenarios. Specifically, for the scenario with 20 clients, the ResNet110 model is first deployed on the clients. When dealing with the data distribution of *QS*, FedCS is 1.86–20.94 higher than other comparison algorithms. When dealing with IID data distribution, FedCS is 0.19 lower than FedDCT but 8.86–18.22 higher than other algorithms. Secondly, the WRN-16-8 model is deployed on the client. When dealing with *QS* data distribution, FedCS is 1.19–20.99 higher than other comparison algorithms. When dealing with *IID* data distribution, FedCS is 0.49–16.16 higher than other comparison algorithms. For the scenario with 40 clients, the ResNet110 model is first deployed on the client. When dealing with *QS* data distribution, FedCS is 1.89–15.12 higher than other algorithms. Similarly, in *IID* data distribution, FedCS is 0.13–24.67 higher than other algorithms. Secondly, the WRN-16-8 model is deployed on the client. When dealing with *QS* data distribution, FedCS is 0.48–28.4 higher than other comparison algorithms. The FedCS is 0.51 lower than FedDCT but 19.6–26.35 higher than other algorithms when dealing with *IID* data distribution. Furthermore, consistent with the results from the Cifar10 dataset, Figure 5 and Figure 6 show that FedCS consistently outperforms the FedDCT algorithm in most cases while also exceeding the performance of FedAvg, FedProx, and FedSplit algorithms.

### 5.5. Model Scalability

**Figure 10:**
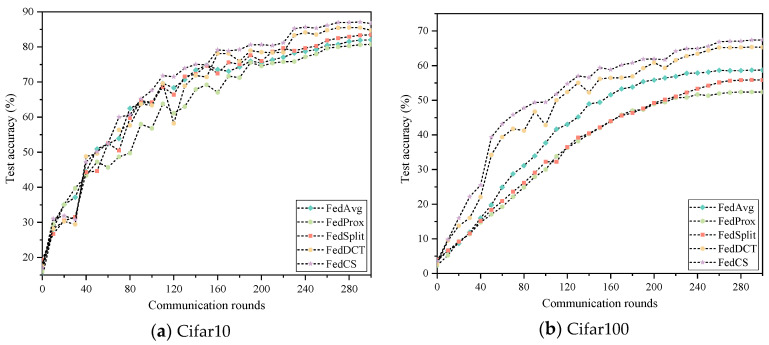
Classification performance of five algorithms on two datasets.

To verify the performance of FedCS applied to depth models of different scales, we deployed the ResNet50 model framework on 20 clients for analysis. For the QS distribution of Cifar10 and Cifar100 datasets, we discuss the performance of FedAvg, FedProx, SplitFed, FedDCT, and FedCS. The experimental results are presented in Table 4 and Figure 10. As shown in Table 4, FedCS achieves excellent performance on both datasets. Specifically, on the Cifar10 dataset, FedCS is 1.96–5.95 higher than other comparison algorithms. On the Cifar100 dataset, FedCS is 2.07–14.99 higher than other comparison algorithms. In addition, as shown in Figure 10, FedCS demonstrates faster convergence and superior classification performance compared to all other algorithms. Furthermore, the experimental results on models of different sizes, such as ResNet-50 and ResNet-110, demonstrate that FedCS is scalable and can maintain high performance across a wide range of model complexities.

## 6. Conclusions

In this paper, a federated collaboration framework with sparse gradients is designed to address the communication overhead, data heterogeneity, and training efficiency of resource-constrained devices in distributed learning environments. First, the model is partitioned across different devices, enabling parallel training on resource-constrained devices for large models. Second, to improve the training efficiency, the sparse gradient strategy is constructed by using position Mask to reduce the data transmission, and the gradient dequantization strategy is introduced to recover the original dense gradient and reduce the negative impact on the model performance. Then, the distance between the client and the global model is assessed with Euclidean distance to measure the performance of each client in the classification task, and then an adaptive weight strategy is designed to assign the client an appropriate aggregate weight. Finally, a new federated collaboration algorithm is designed by combining the sparse gradient quantization method with the adaptive weight strategy. The performance of the proposed algorithm is evaluated using different network models and datasets. Experimental results show that on Cifar10 datasets, our method improves training time by about 35% and accuracy by about 13%. On the Cifar100 dataset, the training time is improved by approximately 8%, while accuracy increases by about 20%.

Although the proposed method efficiently supports distributed training on resource-constrained devices, there are still some directions worth further research:

(1) Because the federated collaborative framework adopts model splitting to assign training tasks to multiple devices, the shared data may expose sensitive user information. Therefore, future work can be combined with existing privacy-preserving technologies, such as differential privacy and multi-party computation, to achieve the protection of transmitted data information. (2) Given the potential for malicious attacks in real-world scenarios, to improve the robustness of the system, methods such as credibility evaluation algorithm and anomaly detection are introduced to identify and isolate untrusted clients and design a mechanism to prevent antagonistic attacks. (3) Considering the communication bottleneck and scale of the federated collaboration framework, future work could focus on optimizing data transmission protocols to reduce communication load, designing more lightweight server-side computation methods, and designing a more simplified weight allocation strategy combined with different types of tasks to reduce the computational burden.

## Figures and Tables

**Table 1:** Experimental parameters and values.

Parameters	Explanation	Value
*R*	Communication rounds	300
*E*	Local training	1
*B*	Local batch	128
*V*	Eval batch	100
*M*	Optimizer momentum	0.9
*η*_0_	Local learning rate	0.1

**Table 2:** Experimental results of five algorithms on the Cifar10 dataset.

Methods	20 Clients				40 Clients			
	ResNet110		WRN-16-8		ResNet110		WRN-16-8	
	*QS*	*IID*	*QS*	*IID*	*QS*	*IID*	*QS*	*IID*
FedAvg	84.97	89.61	90.13	92.13	77.60	82.25	84.35	87.86
FedProx	81.14	84.64	86.61	88.75	73.07	77.63	79.32	82.39
FedSplit	86.29	89.05	89.17	91.82	77.73	81.26	83.14	86.70
FedDCT	86.54	**93.45**	93.89	**95.87**	79.63	90.47	91.25	94.44
FedCS	**87.07**	93.40	**94.16**	95.82	**82.04**	**90.71**	**91.28**	**95.12**

**Table 3:** Experimental results of five algorithms on the Cifar100 dataset.

Methods	20 Clients				40 Clients			
	ResNet110		WRN-16-8		ResNet110		WRN-16-8	
	*QS*	*IID*	*QS*	*IID*	*QS*	*IID*	*QS*	*IID*
FedAvg	59.75	63.90	68.44	72.51	45.97	48.14	54.35	57.09
FedProx	49.39	54.54	58.16	63.78	38.49	41.33	46.73	50.34
FedSplit	59.03	62.53	67.12	70.75	43.59	46.89	53.45	56.47
FedDCT	68.47	**72.95**	77.96	79.45	61.79	65.87	74.65	**77.20**
FedCS	**70.33**	72.76	**79.15**	**79.94**	**63.68**	**66.00**	**75.13**	76.69

**Table 4:** Experimental results of five algorithms on two datasets.

Methods	Cifar10	Cifar100
FedAvg	82.05	58.73
FedProx	80.72	52.39
FedSplit	83.46	55.86
FedDCT	84.71	65.31
FedCS	**86.67**	**67.38**

## Data Availability

The data that support the findings of this study are available from the corresponding author upon reasonable request.
